# Exploring health stakeholders' perceptions on moving towards comprehensive primary health care to address childhood malnutrition in Iran: a qualitative study

**DOI:** 10.1186/1472-6963-9-36

**Published:** 2009-02-23

**Authors:** Sara Javanparast, John Coveney, Udoy Saikia

**Affiliations:** 1 Discipline of Public Health, Flinders University, Faculty of Health Sciences, Flinders University, Adelaide, SA, Australia; 2 Department of Population and Environment Management, School of Geography, Faculty of Social Sciences, Flinders University, Adelaide, SA, Australia

## Abstract

**Background:**

Due to the multifaceted aspect of child malnutrition, a comprehensive approach, taking social factors into account, has been frequently recommended in health literature. The Alma-Ata declaration explicitly outlined comprehensive primary health care as an approach that addresses the social, economic and political causes of poor health and nutrition.

Iran as a signatory country to the Alma Ata Declaration has established primary health care since 1979 with significant progress on many health indicators during the last three decades. However, the primary health care system is still challenged to reduce inequity in conditions such as child malnutrition which trace back to social factors. This study aimed to explore the perceptions of the Iranian health stakeholders with respect to the Iranian primary health care performance and actions to move towards a comprehensive approach in addressing childhood malnutrition. Health stakeholders are defined as those who affect or can be affected by health system, for example health policy-makers, health providers or health service recipients.

**Methods:**

Stakeholder analysis approach was undertaken using a qualitative research method. Different levels of stakeholders, including health policy-makers, health providers and community members were interviewed as either individuals or focus groups. Qualitative content analysis was used to interpret and compare/contrast the viewpoints of the study participants.

**Results:**

The results demonstrated that fundamental differences exist in the perceptions of different health stakeholders in the understanding of comprehensive notion and action. Health policy-makers mainly believed in the need for a secure health management environment and the necessity for a whole of the government approach to enhance collaborative action. Community health workers, on the other hand, indicated that staff motivation, advocacy and involvement are the main challenges need to be addressed. Turning to community stakeholders, greater emphasis has been placed on community capabilities, informal link with other social sectors based on trust and local initiatives.

**Conclusion:**

This research provided a picture of the differences in the perceptions and values of different stakeholders with respect to primary health care concepts. The study suggests that a top-down approach, which still exists among health policy-makers, is a key obstacle that delays, and possibly worse, undermines the implementation of the comprehensive strategy codified by the Alma-Ata Declaration. A need to revitalise primary health care to use its full potential and to combine top-down and bottom-up approaches by narrowing the gap between perceptions of policy makers and those who provide and receive health-related services is crucial.

## Background

The definition of child malnutrition recently shifted from being a biological deficiency of micro and macro nutrients to a broader sense which includes social, environmental and economic determinants [1,2]. A well-developed body of theoretical work, supported by substantial empirical research, links social determinants to the nutritional status of children. The conceptual framework pioneered by UNICEF is amongst the most well-known that illustrates an in-depth inter-relationship between determinants of child malnutrition at different levels [3]. There is also a considerable body of empirical research demonstrating the link between nutritional status of children and specific social determinants such as women's status, food security, and national income [4-8].

Due to the multifaceted aspect of child malnutrition, a comprehensive approach which incorporates preventive and health promotion activities and addresses the social and environmental determinants of childhood malnutrition, has been frequently recommended in health literature. As part of this preventive approach, strengthening of health systems, ideally based on primary health care, is considered to be the route to tackle the root causes of poor nutrition, including in non-health sectors. The fundamental concept of comprehensive primary health care was noted three decades ago in the Alma Ata Declaration and explicitly outlined strategies which would respond appropriately to the current health and nutrition needs, and also addresses the social determinants of poor health and nutrition [9]. The Alma Ata's definition of primary health care incorporates community-based approaches to health care, intersectoral action, citizen participation, coordination of care, equity in access to care, appropriate human resource development, social and cultural suitability and publicly-funded services free to all at the point of delivery. Programs built on the Alma-Ata Declaration tended to define health as not the mere absence of illness but embraced a broader, more integrated and holistic view of health. However, after the Alma Ata Declaration, primary health care has been looked at two different ways: PHC as the first level of contact between health system and client, and PHC as a comprehensive approach which includes preventive and health promotion activities as well as citizen participation and intersectoral action.

As a signatory country to the Alma Ata Declaration, a primary health care approach has been employed in Iran since 1979 as the leading strategy for attaining health gain and equity. Many nutrition-related programs have been implemented through the primary health care network, some of which, such as elimination of iodine deficiency disorder via salt iodisation or breastfeeding promotion, have had significant rates of success [10]. Nevertheless, the Iranian primary health care has been concerned to address the high prevalence and unequal distribution of under-nutrition among children. The prevalence rate of chronic malnutrition (stunting) varies widely from 38.1% in the most disadvantaged province to 6.8% in the well-off province [11]. In other words, it seems that the primary health care approach, as identified in the Alma Ata Declaration, has not been sufficiently implemented to address the social determinants of poor health and nutrition and to secure equity in child's nutritional wellbeing. This gap raised the central question: "what are the main constraints and how can the Iranian primary health care system move towards comprehensive approach and hence attain success in addressing child malnutrition?"

To provide advocacy for the implementation of a primary health care approach, an understanding of the perceptions of stakeholders at different levels needs to be explored. Although many studies in the literature stress the importance and magnitude of malnutrition problem, very few studies deal with an approach explaining the perceptions, values and interests of stakeholders and the extent to which perceptions fit the original concept of comprehensive approach, proposed by international health organizations [12]. This study examined the health system's opportunities, barriers and potential remedies from the viewpoint of health stakeholders.

## Methods

The present study examined the Iranian primary health care system via a stakeholder analysis approach. Grimble et al [[13], p. 3–4] define stakeholder analysis as "an approach for understanding a system by identifying the key actors or stakeholders in the system, and assessing their respective interest in that system". To assess the Iranian stakeholders' perspectives, a field work was undertaken in Iran using qualitative research methods. Qualitative methods are important research approach for identifying problems and issues from the point of view of people involved [14].

Three groups of participants who were involved in the Iranian primary health care were recruited including health policy-makers, health providers and community members. Policy makers included the head or senior expert of the primary health care and nutrition division at national and two selected provinces. Health providers were those who worked as rural community health workers, called *Behvarz*, and community stakeholders comprised mothers with children under age five. Purposeful sampling was used to select policy makers who were information-rich cases [15,16]. Health providers and mothers were selected according to a purposeful and convenience sampling process based on easiest access at the scheduled time in the rural health house where data was collected. Involving a diverse range of stakeholders in the study was invaluable in enabling the researchers to gather different points of view with regards to an identified problem and to reveal conflicts between various stakeholders [17].

Face to face interview and focus group were used for data collection. National and provincial policy makers were interviewed individually in order to allow for meaningful dialogue. Focus groups were selected for the rural health providers and community members to allow participants to interact with each other as well as with the moderator, which can be helpful in terms of increasing the data quality. Ten interviews with the national and provincial health policy-makers, two focus groups (one in each province) with the health providers and 10 focus groups (five in each province) were held with mothers. Interviews and focus groups were conducted by the first author, using a semi-structured interview schedule and topic guide respectively.

Participants were asked a set of questions concerning primary health care as an approach to address childhood malnutrition and perceived barriers and opportunities to the implementation of a primary health care approach within the Iranian health system. Interviews were recorded with the consent of participants. Transcribed materials were then translated to English by the first researcher whose first language was the same as the interviewees. In order to decrease the potential for translation error, all audio-tapes were checked against the transcribed text by the researcher. After transcription, data interrogation comprised two steps: 1) descriptive analysis; allowed understanding of the context of the interview/focus group by breaking down the data and generating themes, 2) interpretative analysis; provided a larger conceptual ideas of the findings [18]. Interviews and focus groups data were coded to comparable categories [19]. Category generation was based on the key concepts in the study questions producing master codes and sub-codes.

After the induction and deduction of relevant themes, a simple description of emerged themes was undertaken, capturing some of the "quotable quotes" which were the actual statements of the participants. The second step, interpretative analysis, tried to make a sense of what the data meant via connecting the findings to the existing literature and the researcher's past experience within the Iranian primary health care.

Ethics approval was granted by the Flinders University of South Australia's Social and Behavioural Research Ethics Committee. Permission has also been granted by the head of district health centres in each province. Confidentiality was assured to all participants and has been maintained throughout this study, as has anonymity.

## Results

A total of 10 health policy-makers, 12 health providers and 60 mothers (see Table 1) were invited to participate in the study. Participants, who were of different ages, had a wide range of experience within the Iranian primary health care system, from four to more than 20 years. Interviews were undertaken between May and July 2006.

**Table 1:** Study Participants

	Ministry of Health	Province I (Mazandaran)	Province II (Golestan)
**Policy maker**	4	3	3
PHC/nutrition manager/expert	(2 managers and 2 senior experts)		
**Health provider**		6	6
**Mother**		30	30

### How the current Iranian primary health care performs in addressing the social determinants of child malnutrition

Themes emerged which contributed to the concept of comprehensiveness in the Iranian primary health care system as understood by the participants. Availability and affordability of primary health care and easy access to maternal and child health services were the central attributes of primary health care identified by almost all interviewees.

The most important thing that our health system has is its availability. Our people can access the health services for free. I do not imagine any place in the region or the world has such a well-structured system. [Person no. 3 = P3, Ministry of Health]

We always bring our child for growth monitoring. Supplements are always available... Behvarzes help us whenever we need them. They are always available, this is very good. [Mother/FG6/Province 2]

The second theme that emerged from the interviews and focus groups as a strength was the contribution of primary health care in improving public, and particularly women's nutritional knowledge. The inclusion of health and nutrition education programs was considered to be an example of a comprehensive primary health care approach which addresses mothers' education as one of the most important underlying determinants of child malnutrition. Ongoing health and nutrition education for mothers, taking the literacy level and practical aspects of the education into account, was an important issue that emerged from the majority of the interviews and focus groups.

In contrast to what is generally believed, I think our health system had less medical and selective approach from the first day of its design. We put education as one of the milestones in nutrition program... I think we were very successful in this area. Actually, we did social-based activities in our system. [P6/Province1]

Members of health provider and parent focus groups concurred with the national and provincial policy makers that nutrition education is one of the highlights of the Iranian health system:

We do have regular health care and nutrition education for mothers before, during and after delivery which increases mothers' knowledge regarding importance of food and nutrition. [Behvarz/FG2/province2]

Behvarz educates us about breastfeeding, and all sorts of foods our child should or shouldn't eat. They also talk about their development stages and growth trend. I learnt a lot from them. [Mother/FG9/province2]

### How the current primary health care system in Iran can move towards a more comprehensive approach to address the social determinants of child malnutrition

The study then attempted to explore the perceptions of stakeholders towards the idea of how the Iranian primary health care is able to act more broadly. A wide range of attitudes were held by various participants. These attitudes varied across the spectrum, from a solely top-down approach, including governmental and political reform, to a more bottom-up approach supporting social mobilization and community participation.

Policy makers took mostly a top-down approach when recommending ways to overcome organizational barriers and to ensure sustainability. For example, establishing a proper health management system was recommended to overcome the managerial problems. Issues such as rapid staff turnover, political conflicts, the lack of staff motivation and position insecurity, misperception and ineffective leadership were pointed to as factors that limit the long-term inter-institutional relations that need to be addressed.

The role of managers is very important in national and provincial approaches within the primary health care system. Unfortunately, due to rapid changes in the managerial levels they just follow acute and flash jobs to be able to demonstrate an acceptable outcome at the end of their short management period. [P8/Province2]

If you review the history of the primary health care in Iran, you see that the health deputies have been changed approximately every six months.... A political approach is followed in the selection of managers and less attention is paid to their scientific capabilities and experiences. [P3/Ministry of Health]

Boosting intersectoral collaboration was the second most strongly articulated issues by the study respondents. From their points of view, only with effective collaboration with other sectors could the primary health care system act comprehensively. Health policy-makers mainly believed in a whole of the government reform in order to manage intra and intersectoral processes and stewardship challenges.

The vision and policies at the highest level of the government should change. In this way, each ministry takes a small part of the responsibility in terms of the nutritional status of people... If this happens, collaboration occurs not only at ministerial levels but also at the most peripheral areas. [P2/Ministry of Health]

Turning to the health providers and community stakeholders, fundamental differences were highlighted in terms of the existing weak points and actions to overcome them. They mainly looked from a more bottom-up window to address the social determinants of child malnutrition. From their perspective, horizontal links between various sectors should not necessarily rely on the government reform. Providing a supportive environment for health providers to build collaborative actions was a point frequently articulated by the participants. From the health providers' perspective:

We are tired of telling our problems. There is nobody to hear our voice and no place to complain. High workload, low wage, pile of forms to be completed, low support and encouragement system for behvarz, and no promotion are all our problems that nobody cares. We are not involved in program planning. Having all these problems, make us feel tired. Nevertheless, we work just for our people's sake. [Behvarz/FG 1/province 1]

Self-reliance and local innovations were emphasised at the community level by strengthening the self-help capabilities of the rural disadvantaged. Mothers believed community development initiatives to be more feasible actions to advocate families in need.

We know everybody in the village... The representatives of the other organisations are present in rural health councils, so with minimum advocacy we are able to work with them and introduce needy women and families to them, because nobody knows families better than we do. [Behvarz/FG2/Province 2]

Community self-helping is a big source of support for families in need. Health house and Behvarz can be more involved in this area because they know everybody, and everybody trusts them. In this way, the help can be organised and spent in the best way to meet people's needs. [Mother/FG8/Province2]

The above quotes demonstrate that local actions are mostly commented on by the community members.

## Discussion

The debate around comprehensive primary health care and between horizontal and vertical, as well as top-down and bottom-up approaches was the major focus in discussions related to primary health care approach after the Alma Ata Declaration. Iran, which has a well-established primary health care system and is a signatory country to the Alma Ata Declaration, is an appropriate case study to investigate how the concept of comprehensiveness has been understood and implemented. The findings of this study demonstrate that some aspects of comprehensive primary health care, such as equitable access to basic health services, are paid more consideration than other considerations, such as citizen participation and bottom-up approaches. The provision of an available and affordable health services, including integrated maternal and child care programs for all segments of the community was believed to be an opportunity by a vast majority of the participants. In health literature, equitable access to primary health care, in terms of geographical distribution and distance to health care providers, is recognised as a characteristic of comprehensive primary health care, a significant contributor to population health in general and an important factor in reducing malnutrition among children, in particular [20]. Apart from physical and financial access, acceptability – which refers to as cultural distance between health system and its users – is also thought to be an important factor [21]. Based on data collected here, it can be argued that health care accessibility in terms of physical, financial and cultural access represents one of the most comprehensive components of the Iranian primary health care.

The narrow perception of the comprehensive primary health care approach can be more clearly understood when the other pillars of the approach, such as community participation, collaborative action and bottom-up approach are taken into account. The Iranian health policy-makers mostly view community participation as a process of community nutrition education which was articulated by the participants as the second most rewarding and comprehensive approach in tackling child malnutrition. The main emphasis of policy makers was on the education of community members, particularly mothers, in order to raise mothers' awareness, with the aim that they will accept and practice what they have been taught. However, given the link between health education, health literacy and health promotion concepts [22-24] which are based on the notion of power transformation and community empowerment, it can be argued that this concept may be viewed in its narrowest sense by the Iranian health policy-makers who recognise people as the recipients of the health and nutrition services. Given that this study employed a stakeholder analysis approach, it was able to compare and contrast the perceptions and values of health stakeholders at different levels. The findings from Iran case study revealed that fundamental differences exist among different stakeholders in the understanding of comprehensive approach and the remedial actions which are required to tackle the root causes of malnutrition and its unequal distribution.

By raising questions with regards to the ways to move towards comprehensive primary health care, policy makers believed that promoting a collaborative action should be seen as part of the national government's stewardship responsibility. High-level managers had mainly a 'whole of the government approach', with special emphasis on the role of national and provincial governments in the provision of opportunities in organisational environments to support collaboration. In this way shared leadership as well as horizontal and vertical links between and within organisations should be established. This approach is also more likely to guarantee sustainability of long term commitment to nutritional well-being.

Health providers and parents at local levels, on the other hand, paid more attention to advocacy, informal links with other social sectors based on trust, self-help and individual relationships. They also stressed self-reliance, knowing their own resources and how and when to use them, to turn to others for support and cooperation. The notion of using local capacities, authorities and initiatives to ensure nutritional well-being was rarely mentioned by the policy makers. In other words, the power of community to participate in the planning and implementation of nutritional programs for their children and to accomplish intersectoral action was underestimated in the Iranian primary health care. This can be considered as a low level of community participation as classified by Ismail [25] from a passive form as the recipient of services to self-mobilization in which people take initiatives independently and have control over decision-making. This perspective seems common in other settings with similar infrastructure to Iran. A study by Tatar in Turkey also concluded the lack of a comprehensive understanding in terms of community participation amongst Turkish health policy-makers [26].

**Figure 1:**
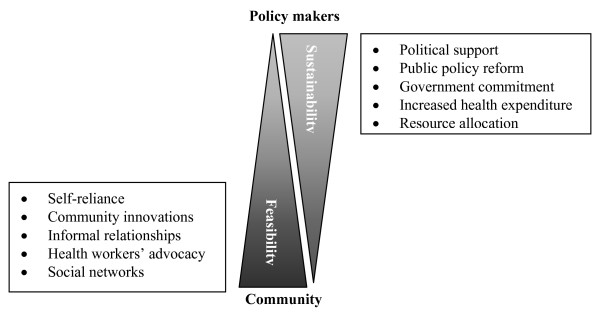
**Perceptions of the Iranian health stakeholders in moving towards comprehensive primary health care in Iran**.

Figure 1 represents differences in the perceptions of the Iranian health stakeholders concerning actions to move towards a comprehensive approach.

It is argued that comprehensive approach develops with engaging the broader community and providing resources for support [27]. Baum [28] also argues the combination of a top-down pressure from policy makers and bottom-up actions from civil society in order to tackle the growing problem of health inequity, which is in her words "cracking the nut of inequity".

The above discussion on the primary health care perspective does not mean that the attempts in the Iranian health sector to implement comprehensive approach to primary health care have been insubstantial. The results of this study has brought several positive points including the overall awareness of the health stakeholders with respect to the importance of social factors influencing childhood malnutrition and high access to affordable health services. However, it is argued that to strengthen the health system, a much better and in-depth understanding of all aspects of comprehensive primary health care followed by careful implementation by those who are involved within the system is crucial.

## Conclusion

It can be concluded that although the principles of the Iranian primary health care have included community participation and bottom-up approaches, they may not be among the priorities of the policy development process. This partly traces back to the policy makers' misperception concerning principal components of comprehensive primary health care. If primary health care is still judged to be a viable approach, a combination of the more sustainable and feasible actions should be analysed thoroughly.

There is a need to revitalise primary health care in Iran to use its full potential. Combining top-down and bottom-up approaches by narrowing the gap between the perceptions of policy makers and those who provide and receive health-related services seems crucial. And to achieve that, perceptual reform of health policy-makers needs to be considered as an important and crucial strategy in revitalizing comprehensive primary health care.

## Competing interests

The authors declare that they have no competing interests.

## Authors' contributions

All authors contributed to the conception and design of this study. SJ collected and analysed the qualitative data via fieldwork with supervision from JC and US. All authors contributed to drafts of this manuscript and approved the version being submitted.

## Pre-publication history

The pre-publication history for this paper can be accessed here:


